# A critique of general allometry-inspired models for estimating forest carbon density from airborne LiDAR

**DOI:** 10.1371/journal.pone.0215238

**Published:** 2019-04-19

**Authors:** Rebecca A. Spriggs, Mark C. Vanderwel, Trevor A. Jones, John P. Caspersen, David A. Coomes

**Affiliations:** 1 Department of Plant Sciences, University of Cambridge, Cambridge, United Kingdom; 2 Department of Biology, University of Regina, Regina, Saskatchewan, Canada; 3 Ontario Ministry of Natural Resources and Forestry, Forest Research and Monitoring Section, Sault Ste. Marie, Ontario, Canada; 4 Faculty of Forestry, University of Toronto, Toronto, Ontario, Canada; University of Hawaii, UNITED STATES

## Abstract

There is currently much interest in developing general approaches for mapping forest aboveground carbon density using structural information contained in airborne LiDAR data. The most widely utilized model in tropical forests assumes that aboveground carbon density is a compound power function of top of canopy height (a metric easily derived from LiDAR), basal area and wood density. Here we derive the model in terms of the geometry of individual tree crowns within forest stands, showing how scaling exponents in the aboveground carbon density model arise from the height−diameter (H−D) and projected crown area−diameter (C−D) allometries of individual trees. We show that a power function relationship emerges when the C−D scaling exponent is close to 2, or when tree diameters follow a Weibull distribution (or other specific distributions) and are invariant across the landscape. In addition, basal area must be closely correlated with canopy height for the approach to work. The efficacy of the model was explored for a managed uneven−aged temperate forest in Ontario, Canada within which stands dominated by sugar maple (*Acer saccharum* Marsh.) and mixed stands were identified. A much poorer goodness−of−fit was obtained than previously reported for tropical forests (R^2^ = 0.29 vs. about 0.83). Explanations for the poor predictive power on the model include: (1) basal area was only weakly correlated with top canopy height; (2) tree size distributions varied considerably across the landscape; (3) the allometry exponents are affected by variation in species composition arising from timber management and soil conditions; and (4) the C-D allometric power function was far from 2 (1.28). We conclude that landscape heterogeneity in forest structure and tree allometry reduces the accuracy of general power-function models for predicting aboveground carbon density in managed forests. More studies in different forest types are needed to understand the situations in which power functions of LiDAR height are appropriate for modelling forest carbon stocks.

## Introduction

Aboveground carbon density (ACD) is an important forest property to map in the context of the global carbon cycle [1–3]. Classically, ACD has been estimated using tree size measurements recorded from networks of forest plots, with generalised or species−specific allometries used to convert field measures of diameter and height into tree biomass estimates, and then into ACD estimates [4, 5]. More recently, methods using remote sensing technologies have been developed to complement these plot networks: airborne or spaceborne LiDAR sensors have proven to be particularly effective for estimating ACD because they provide detailed information about forest structure, which is in turn closely related to ACD [6].

There is currently much interest in developing a general method for predicting ACD from LiDAR [7, 8]. A common approach has been to estimate ACD in field plots and then use regression to relate these measurements to various LiDAR metrics [9]. This approach can deliver accurate estimation models within sampling regions, but the models lack physical underpinnings because they are purely empirical. Consequently, they either need to be re-parameterized for each new site, or generalised by estimating how parameters vary geographically. Asner and Mascaro [8] have developed a General Model (henceforth AM−GM) for predicting ACD, which uses measures of the top canopy height derived from LiDAR (*H*_*L*_), along with local relationships predicting basal area (*B*_*P*_) and basal−area−weighted mean wood density $\left({\overset{-}{\rho }}_{P}\right)$:

$$ACD=a{H_{L}}^{b_{1}}{B_{P}}^{b_{2}}{{\overset{-}{\rho }}_{P}}^{b_{3}}$$ (1)

where *a*, *b*_*1*_, *b*_*2*_ and *b*_*3*_ are parameters estimated by regression using the log−transformed function. Note that subscript *L* denotes a LiDAR−based measurement, and subscript *P* a plot−based measurement. Asner and Mascaro [8] argue that this model is analogous to the allometric formula used to calculate an individual tree’s biomass from its height *H*_*i*_, diameter *D*_*i*_ and wood density *ρ*_*i*_ measurements, namely $aH_{i}^{b}D_{i}^{c}\rho_{i}^{d}$ where *a*, *b*, *c* and *d* vary with forest type [10] and *i* denotes measurements on an individual tree. Fitting the AM−GM to data from four contrasting tropical forests, Asner et al. [7] found that a single, universally fitted relationship reduced model accuracy by no more than 1% relative to regional−specific models. Furthermore, the accuracy was only slightly diminished by replacing plot−level measurements of *B*_*P*_ and ${\overset{-}{\rho }}_{P}$ with regional averages and, as a result, the major benefit of their approach is that it requires less additional field data to calibrate than traditional regressions [11].

A key reason why the AM−GM has worked well, where it has, is that basal area and top-of-canopy height were closely correlated in the forests investigated. Asner and Mascaro [8] showed that–for the four tropical forests studied–the AM−GM could be calibrated simply by generating a local relationship estimating *B*_*P*_ from LiDAR and finding a regional ${\overset{-}{\rho }}_{P}$ estimate. Others have questioned the generality of the approach [12,13]. In some forest types the correlation between forest height and basal area is weak, especially for mature stands. In these situations two stands can have the same top-of-canopy height, but quite different basal area [14,15].

The problem is that the carbon density of a plot is obtained by summing the biomass of individual trees, but because a tree’s biomass is non-linearly related to its dimensions (height, stem diameter), this summation is only exact under certain conditions that we explain below. Although Asner et al. [7] did not claim that the AM−GM could be applied outside the tropics, testing the accuracy of the model across different forest types is important to understanding the applicability and limitations of the general model. For example, tropical and temperate forests have contrasting size structures: rain forests contain shade-tolerant species that develop a dense understory beneath the upper canopy (i.e. stands contain many small trees and few large trees), while temperate forests often lack dense understories and can have unimodal size-frequency distributions [16]. Perhaps for this reason the AM−GM had low goodness−of−fit when applied to broadleaf and coniferous forests in the USA [13], but this has yet to be evaluated critically. Vincent et al. [12] suggest that forests should first be delineated into homogenous regions with respect to the relationships between forest structure and LiDAR data to improve model performance. Unfortunately, this requirement would severely limit the generality of the model.

The aim of this study is to derive the AM−GM from first principles using the geometry of individual trees and, by doing so, to improve understanding of when the AM−GM is likely to yield accurate predictions (i.e., have high goodness−of−fit when applied to data from the field and from LiDAR scanners). Our individual−tree−based general model (ITB−GM) has the same functional form as the AM−GM (1), but its parameters are derived from individual tree allometries and other assumed scaling relationships. We fit the AM−GM to data from an uneven−aged forest in central Ontario, Canada and compare the parameter estimates with those obtained from tree−based measurements using the ITB−GM. By doing so, we explore why the AM−GM has poor predictive ability in this temperate forest. We then examine whether fitting separate models for two forest types within the Canadian dataset leads to significant improvements in goodness−of−fit. Finally, we outline forest conditions that determine the accuracy of the AM−GM.

### Theory: An individual−tree−based general model

Consider a tree with stem diameter *D*_*i*_ (in cm), height *H*_*i*_ (in m), vertically projected crown area *C*_*i*_ (in m^2^) and wood density *ρ*_*i*_ (in g/cm^3^) growing in a plot with an area *A*_*P*_ (in ha). The tree’s aboveground biomass can be modelled as $a_{1}{\pi D_{i}}^{2}H_{i}\rho_{i}$ where *a*_1_ is a species−specific coefficient that depends on crown and stem form. The total aboveground biomass of the plot is found by summing the biomasses of all *N*_*P*_ trees in the plot. ACD is calculated by dividing this biomass value by *A*_*P*_ and multiplying by carbon content *a*_0_ (typically 0.5):

$$ACD=a_{2}\sum_{i=1}^{N_{p}}a_{0}a_{1}{{\rho_{i}D}_{i}}^{2}H_{i}$$ (2)

where *a*_2_ = *π*/*A*_*P*_. For ease of presentation, the limits of summations are dropped in subsequent equations, but remain the same throughout.

Assuming that a tree’s height is related to its diameter by a power function $\left(H_{i}=a_{H}{D_{i}}^{k_{H}}\right)$, we get:

$$ACD=a_{2}\sum a_{0}a_{1}a_{H}\rho_{i}{D_{i}}^{2+k_{H}}$$ (3)

We can use individual tree heights and crown areas to estimate the average top canopy height *H*_*P*_: this is calculated by summing the crown top height of all trees in the plot, weighted by their crown areas, *H*_*P*_ = (∑*a*_3_*C_i_H_i_*)/*C_P_* where the canopy area of the plot is $C_{P}=\sum_{j=1}^{N_{p}}C_{j}$ and *a*_3_ is a multiplier that takes into account that the average height of each tree’s crown is some fraction of that tree’s maximum height [15]. Assuming that crown area is also a power function of stem diameter $\left(C_{i}=a_{C}D_{i}^{k_{C}}\right)$, and that $H_{i}=a_{H}D_{i}^{k_{H}}$ as before, we get:

$$H_{P}=\frac{1}{C_{P}}\sum a_{3}a_{H}{a_{C}D}_{i}^{k_{C}+k_{H}}$$ (4)

Our aim is to substitute (4) into (3) to remove the *D*_*i*_ terms, so that ACD is expressed in terms of *H*_*P*_, *B*_*P*_ and ${\overset{-}{\rho }}_{P}$. However, this is not straightforward for two reasons. The first problem is that *a*_0_, *a*_1_, *a*_3_, *a*_*H*_, *a*_*C*_ and *ρ* are inside the summations, but cannot necessarily be moved outside the summations because they are species−specific variables. As an approximation, we represent them by tree−volume−weighted mean values and take them outside of the summation [12] to give $ACD\approx a_{2}{\overset{-}{a}}_{0}{\overset{-}{a}}_{1}{\overset{-}{a}}_{H}{\overset{-}{\rho }}_{P}\sum {D_{i}}^{2+k_{H}}$ and $H_{P}\approx {\overset{-}{a}}_{3}{\overset{-}{a}}_{H}{\overset{-}{a}}_{C}{C_{P}}^{-1}\sum D_{i}^{k_{C}+k_{H}}$. The second problem is that *D*_*i*_ is raised to different exponents inside the two summations (except when *k*_*C*_ = 2). In order to progress, we need to assume that the two summations are themselves related by a scaling function: $\sum D_{i}^{2+k_{H}}\approx a_{D}{\left(\sum D_{i}^{k_{C}+k_{H}}\right)}^{k_{D}}$; we call this the volume summation scaling relationship. The canopy area can be substituted with basal area by assuming a second scaling function: $C_{P}\approx {a_{B}B_{P}}^{k_{B}}$; we call this the canopy area scaling relationship. Making these substitutions, we obtain an individual-tree-based general model (ITB−GM):

$$ACD\approx a_{4}{\left(H_{P}\right)}^{k_{D}}({B_{P})}^{{k_{D}k}_{B}}{\overset{-}{\rho }}_{P}\;where\;a_{4}\approx {{\overset{-}{a}}_{0}{\overset{-}{a}}_{1}{\overset{-}{a}}_{H}a_{2}a_{D}(\frac{a_{B}}{{\overset{-}{a}}_{3}{\overset{-}{a}}_{H}{\overset{-}{a}}_{C}})}^{k_{D}}$$ (5)

This equation is analogous to the AM−GM, given in (1), with *a* = *a*_4_, *b*_1_ = *k*_*D*_, *b*_2_ = *k*_*D*_*k*_*B*_ and *b*_3_ = 1, but it has more parameters and so is less powerful for predictions.

Our derivation based on tree allometries shows that certain parameters in the AM−GM depend on the exponents of the volume scaling relationship and canopy area scaling relationship. It is important to realise that it would be impossible to derive a function having the form of the AM−GM unless these scaling relationships are valid. In the Supporting Information (S1 Text) we show that these relationships are mathematically valid when tree sizes are precisely power-law or Weibull distributed. If the tree size distributions of all stands across a forest follow one of these functions (with identical parameters), the summation can be replaced by an integral that has an analytical solution. Specifically, if a large number of diameters (D_i_,…D_N_) are drawn from *p*(*D*) = *αD*^−*β*^ (where *α* is a normalising constant), then a given power function summation can be approximated by:

$$\sum_{i=1}^{N}{D_{i}}^{\gamma }\approx N\int_{D_{min}}^{D_{max}}D^{\gamma }p(D)dD=\alpha N\int_{D_{min}}^{D_{max}}D^{\gamma -\beta }\;dD$$ (6)

which can in turn be solved to give:

$$\sum_{i=1}^{N}{D_{i}}^{\gamma }\approx \frac{\alpha N}{\gamma -\beta +1}[{D_{max}}^{\gamma -\beta +1}-{D_{min}}^{\gamma -\beta +1}]$$ (7)

A similar property holds for a Weibull distribution of tree diameters [S1 Text]. If the power or Weibull distribution is identical across stands, it can be shown that *k*_*D*_ = *k*_*B*_ = 1 and *a*_*D*_ and *a*_*B*_ are both predictable.

We now compare the performance of the AM−GM and ITB−GM using data from a temperate forest, to gain a better understanding of when these models are appropriate for estimating ACD from LiDAR data.

## Materials and methods

### Study area and inventory dataset

We used datasets from Haliburton Forest and Wildlife Reserve in central Ontario, Canada (45°13’N, 78°35’W). The forest is managed using selection silviculture and consists mostly of uneven−aged stands [17]. Sugar maple (*Acer saccharum* Marsh.) is the most prevalent species, but a number of other species are common, including eastern hemlock (*Tsuga canadensis* (L.) Carrière), balsam fir (*Abies balsamea* (L.) Mill.) and American beech (*Fagus grandifolia* Ehrh.). There were 154 circular plots inventoried across the forest each with an area of 2500 m^2^. The plot locations were chosen to stratify the variation across the forest. The stem diameters of all trees with a stem diameter equal to or greater than 8 cm were recorded along with their species identity. The plots were randomly split into a calibration (114 plots) and a validation dataset (40 plots). The calibration dataset was used for fitting the models and relationships, whilst the validation dataset was reserved for assessing model performance.

ACD was estimated for each plot using species−specific allometric equations developed for Canadian inventories, which relate stem diameter to aboveground tree biomass [18, 19]. Species−specific equations were used for the seven most prevalent species and then generic conifer and broadleaf equations were used for all remaining species (~ 17% of total trees). The individual tree aboveground biomasses were summed for each plot and converted to a per hectare estimate; this aboveground biomass estimate was then multiplied by the carbon content of wood (0.5; [20]) to estimate ACD. Wood density estimates were extracted from [21] and represent the oven dry mass divided by green volume. To parameterise the LiDAR models (AM−GM and ITB−GM), wood density was summarised as a volume−weighted average for each plot $\left({\overset{-}{\rho }}_{P}\right)$. Finally, we succinctly described the tree size distribution of each plot by calculating the quadratic mean diameter (QMD) as $200\sqrt{{A_{P}B}_{P}/\left({\pi N}_{P}\right)}$, and by fitting a Weibull distribution to the list of stem diameters.

### Airborne LiDAR

The LiDAR data were collected using an Optech ALTM 3100 four−pass system flown in August 2009 (altitude = 1500 m; pass overlap = 30%; pulse density = 2 pulses/m^2^). The dataset consisted of x, y and z coordinates (converted to the height above the ground by subtracting the digital elevation model) with up to four returns recorded from a single pulse. We used discrete−return airborne LiDAR data clipped in ArcGIS 10 to overlay the inventoried plots, which had been georeferenced to sub−metre accuracy using a Trimble Geo XH 6000. The LiDAR metrics used in the analyses were *H*_*L*_ and gap fraction (*G*_*L*_) (Table 1). We split each plot into 1 m by 1 m tiles and extracted the maximum recorded height of pulses in each of those tiles. *H*_*L*_ was calculated as the mean of the tile heights that were recorded at 2 m and above, which excluded the tiles where LiDAR pulses were not intercepted by the canopy. *G*_*L*_ was calculated as the proportion of first returns recorded at a height less than 2 m above the ground.

**Table 1:** Definitions of all terms and parameters in the AM−GM and ITB−GM.

Term	Definition	Units
**Lidar metrics**		
*H*_*L*_	Top canopy height	*m*
*G*_*L*_	Gap fraction	No units
**Tree level measurements**		
*ρ*_*i*_	Wood density	*Mg m*^−3^ ≡ *g cm*^−3^
*D*_*i*_	Diameter	*cm*
*B*_*i*_	Basal area	*m*^2^
*H*_*i*_	Stem height	*m*
*C*_*i*_	Crown area	*m*^2^
**Plot based measurements**		
*ACD*	Aboveground carbon density	*Mg C ha*^−1^
${\overset{-}{\rho }}_{P}$	Mean wood density (weight by relative abundances of species)	*Mg m*^−3^ ≡ *g cm*^−3^
*C*_*P*_	Canopy area $\left(C_{P}=\sum_{j=1}^{N}C_{j}\right)$	*m*^2^
*A*_*P*_	Plot area	*ha*
*N*_*P*_	Total number of stems in a plot	No units
*H*_*P*_	Average top canopy height	*m*
*B*_*P*_	Basal area	*m*^2^ *ha*^−1^
*QMD*	Quadratic mean diameter	*cm*
**Model parameters**		
*a*_*0*_	Carbon content of trees	
*a*_1_	Coefficient related to crown and stem form	
*a*_2_	Factor scaling stem diameter to plot level basal area	
*a*_3_	Average crown height as a proportion of tree height	
*a*_4_	Coefficient in final ACD equation which amalgamates other coefficients	
${\overset{-}{a}}_{0},{\overset{-}{a}}_{1},{\overset{-}{a}}_{3}$	Means of *a*_0_, *a*_1_, and *a*_3_, weighted by tree volumes	
*a*_*H*_, *k*_*H*_	Coefficient and exponent of scaling relationship between stem diameter and height (H−D)	
*a*_*C*_, *k*_*C*_	Coefficient and exponent of scaling relationship between stem diameter and crown area (C−D)	
*a*_*D*_,*k*_*D*_	Coefficient and exponent of scaling relationship between two summations of stem diameter raised to different powers (volume scaling relationship)	
*a*_*B*_, *k*_*B*_	Coefficient and exponent of scaling relationship between canopy area and basal area (canopy area scaling relationship)	

### Forest types from aerial photography

The study area was classified into two forest types using aerial photographs (captured by an ADS52 Leica camera). The photographs were manually delineated into 42 forest types using standard methods developed by Ontario’s Forest Resources Inventory programme [22]. We reduced the number of forest types to just two according to estimated species composition: stands dominated by sugar maple, and mixed stands that contained a significant coniferous component alongside sugar maple (see [23] for further details on the method used).

### Fitting the AM−GM to the Canadian data

The log−transformed AM−GM was fitted using least squares regression to ACD measured in the calibration plots:

$$\text{ln}\;ACD=\text{ln}\;a\;+b_{1}\;\text{ln}\;H_{L}+b_{2}\;\text{ln}\;B_{P}+b_{3}\;\text{ln}\;{\overset{-}{\rho }}_{P}$$ (8)

Predicted ACD values included a *e*^*MSE*/2^ multiplier (where MSE is the mean square error of the regression) to correct for a bias introduced by the log transformation [24]. *B*_*P*_ and ${\overset{-}{\rho }}_{P}$ were estimated from relationships with LiDAR so that the model could be used to predict ACD outside of the measured plots. We compared the accuracy of models based on LiDAR estimates of *B*_*P*_ and ${\overset{-}{\rho }}_{P}$ against models where *B*_*P*_ and ${\overset{-}{\rho }}_{P}$ were ground measurements, to quantify the loss in accuracy as a result of this estimation approach.

We measured the accuracy of the 40 validation plot predictions of the ACD model and the *B*_*P*_ and ${\overset{-}{\rho }}_{P}$ equations using the coefficient of determination (R^2^):

$$R^{2}=1-\frac{\sum_{j=1}^{40}{\left(P_{j}-O_{j}\right)}^{2}}{\sum_{j=1}^{40}{\left(O_{j}-\overset{-}{O}\right)}^{2}}$$ (9)

where the observed and predicted value for each plot is denoted by *O*_*j*_ and *P*_*j*_, respectively, and the overall mean observed value is denoted by $\overset{-}{O}$. We compared model support using the Akaike information criterion (AIC) where *k* is the number of estimated parameters and *L* is the maximised likelihood function:

AIC=2k-2ln(L) (10)

We also calculated the percentage root mean square error (% RMSE) which is normalised using the mean of the observed values:

$$\%\;RMSE=\frac{100}{\overset{-}{O}}\sqrt{\frac{\sum_{j=1}^{40}{\left(P_{j}-O_{j}\right)}^{2}}{40}}$$ (11)

### Estimating the parameter values of the ITB−GM from tree level information

Exponents *k*_*B*_ and *k*_*D*_ of the ITB−GM equation $\left(ACD\approx a_{4}{H_{L}}^{k_{D}}{B_{P}}^{{k_{D}k}_{B}}{\overset{-}{\rho }}_{P}\right)$ are derived from the volume summation and canopy area scaling relationships. To estimate these, we first estimated allometric scaling exponents *k*_*H*_ and *k*_*C*_ from dimensional measurements of 5436 trees at a site 230 km from the study area [25]. We calculated the relative abundances of species within the 114 calibration plots [S1 Table], then drew 500 trees at random from the height and crown radius dataset such that the species composition of the sample was the same as observed in the plots. Power functions were then fitted to the height vs. diameter and crown area vs. diameter relationships for these 500 trees. The fitted power functions gave values for *k*_*H*_ and *k*_*C*_ that were representative of the species composition in our study area. Exponent *k*_*D*_ (of the volume scaling relationship) was estimated by calculating $log(\sum D_{i}^{2+k_{H}})$ and $log(\sum D_{i}^{k_{C}+k_{H}})$ for each of the 114 calibration plots, and then fitting a power function through these data. Similarly, exponent *k*_*B*_ of the canopy area scaling relationship was estimated by calculating log(*C*_*P*_) and log(*B*_*P*_) for each of the 114 calibration plots, and then fitting a power function through these data. Theoretically, *a*_4_ in the ITB−GM could be calculated as ${\overset{-}{a}}_{0}{\overset{-}{a}}_{1}{\overset{-}{a}}_{H}a_{2}a_{D}{{a_{B}}^{k_{D}}{(\overset{-}{a}}_{3}{\overset{-}{a}}_{H}{\overset{-}{a}}_{C})}^{{-k}_{D}}$ but in practice several of these variables are hard to determine. For this reason, *a*_4_ was estimated by linear regression: we fit log(*ACD*) as a linear function of log(*H*_*L*_), log(*B*_*P*_) and $log({\overset{-}{\rho }}_{P})$ with the coefficients associated with these explanatory variables fixed at the values calculated from individual−tree−based information, such that only *a*_4_ was estimated.

### Testing whether forest type information improves model accuracy

To explore whether incorporating forest type information improved the predictive power of the estimation model, we split the plots into sugar maple and mixed stands using the aerial photographs and repeated the same procedures as above for fitting AM−GM and ITB−GM. Forest type was incorporated into both of these models and into the equations estimating *B*_*P*_ and ${\overset{-}{\rho }}_{P}$ from *H*_*L*_ and *G*_*L*_.

## Results

### Predicting temperate forest biomass using general power-law models

**Fig 1:**
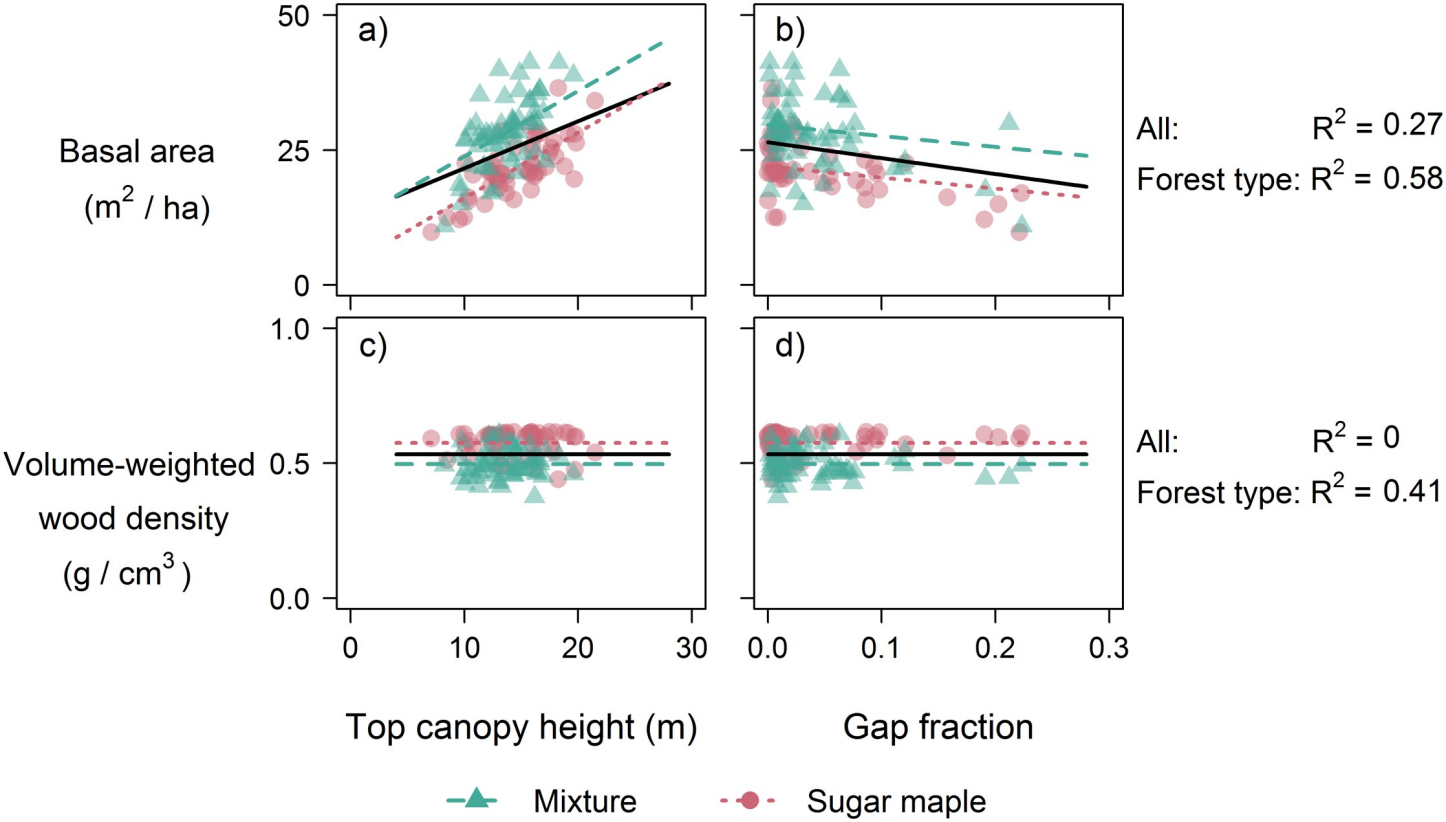
Relationships between field-measured biophysical properties (basal area B_P_ and wood density ${\overset{-}{\rho }}_{P}$) and LIDAR metrics (top-of-canopy height H_L_ and gap fraction G_L_). The lines are predictions from multiple regression analyses of data from all sites (solid), mixed stands (dashed) and sugar maple (dotted). For panels (a) and (c), the predicted lines are obtained by holding G_L_ constant at its mean value, whilst for panels (b) and (d) the value of H_L_ was held at its mean value.

**Fig 2:**
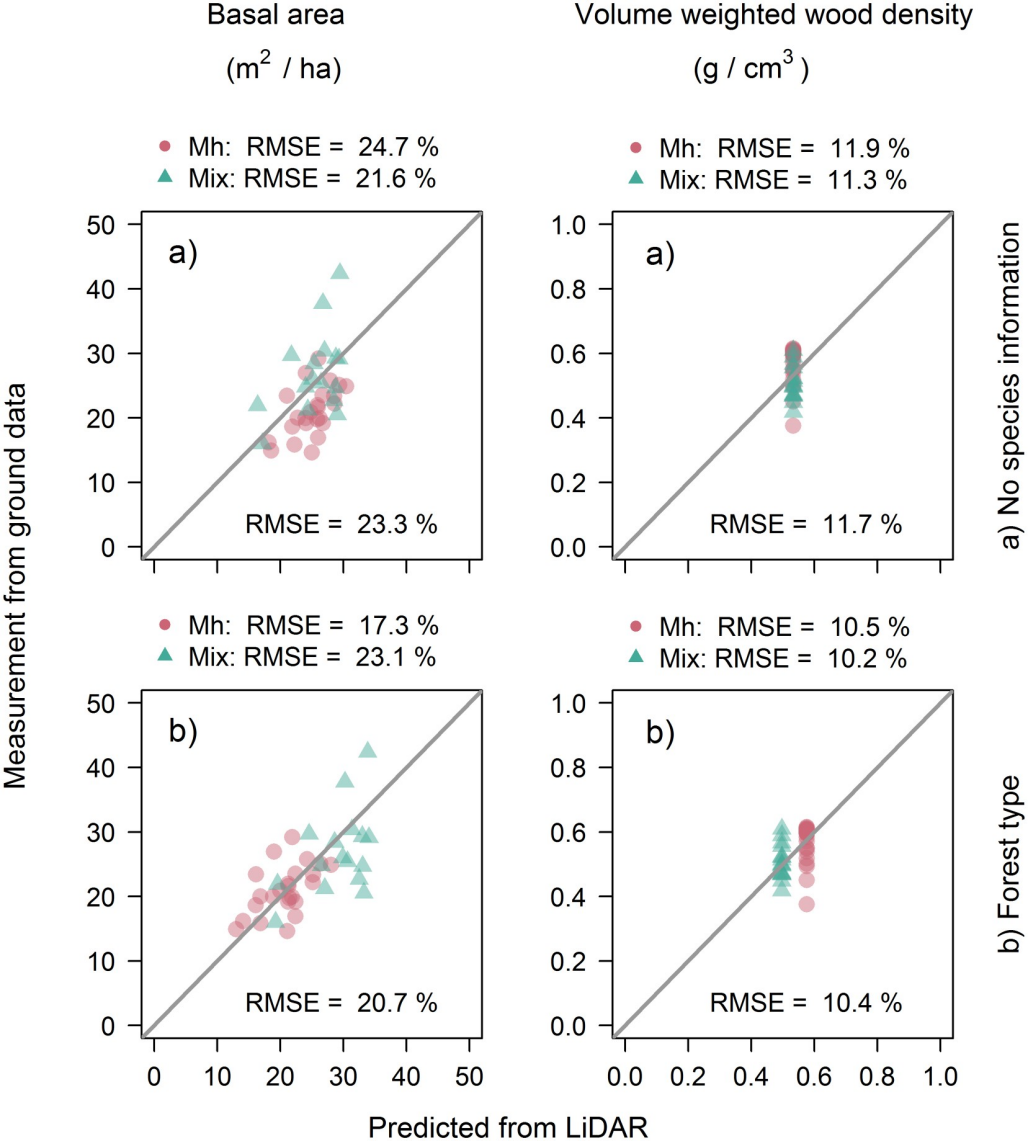
Predictions made for the validation plots by multiple-regression models for basal area (left column) and volume weighted wood density (right column) with: a) no species information and b) forest types.

**Fig 3:**
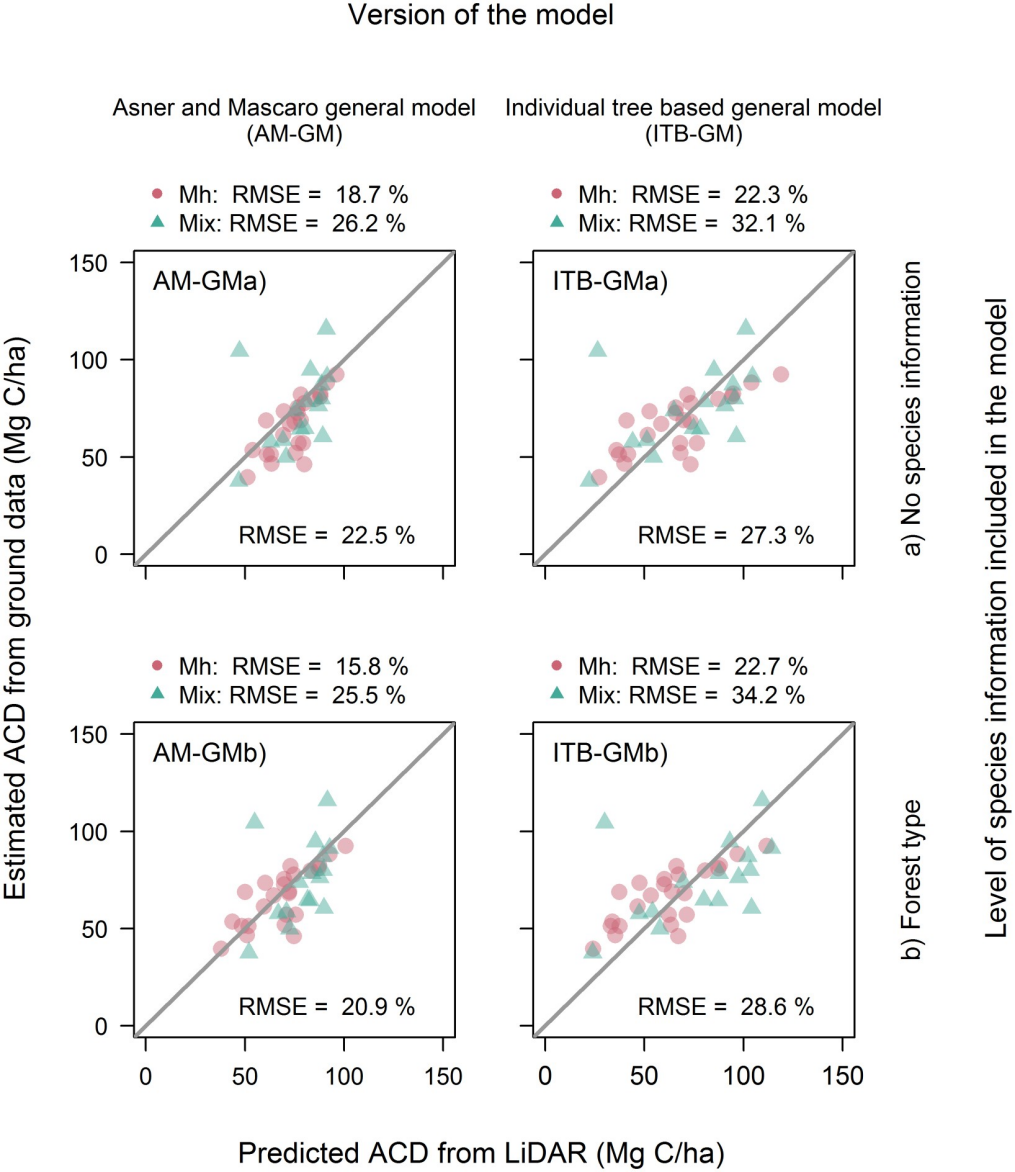
LiDAR vs ground estimated ACD in 40 validation plots, where LiDAR estimates are based on Asner and Mascaro’s general model (AM−GM; first column) and the individual tree based general model (ITB−GM; second column). The first row gives the fit of the AM−GM and ITB−GM to the 40 validation plots (AM−GMa and ITB−GMa) and the second row gives the fit of the models fitted separately to 24 sugar maple and 16 mixed−species stands (AM−GMb and ITB−GMb). The overall RMSE value for each model version is given in the bottom right corner of the plot and the individual RMSE for the sugar maple (Mh) plots and mixture plots (Mix) are given above the plot.

A summary of the coefficients and goodness−of−fit estimates of the AM−GM (1) fitted to the Canadian temperate forest dataset are provided in Table 2. The coefficient of the $log({\overset{-}{\rho }}_{P})$ term was not significantly different from zero, so we set the power (*b*_*3*_) to 1 to match the ITB−GM. The resulting model performed relatively poorly, as the R^2^ of the fit to the validation plots was only 0.18. Fitting the model with ground−measured *B*_*P*_ and ${\overset{-}{\rho }}_{P}$ increased the R^2^ to 0.41, but unfortunately *B*_*P*_ was poorly predicted from LiDAR estimates of *H*_*L*_ and *G*_*L*_ (R^2^ = 0.09; Table 3), and *${\overset{-}{\rho }}_{P}$* was unrelated to the LiDAR metrics (Figs 1 and 2). As a result, we found that ACD could be estimated using the AM−GM with relatively low accuracy (22.5% RMSE; equivalent to a RMSE of 15.7 Mg C ha^−1^; Fig 3).

**Table 2:** Aboveground carbon density (ACD) estimation models fit to a Canadian temperate forest dataset containing sugar maple and mixed broadleaf-conifer stands. Parameters shown in bold were estimated from individual tree data, while all other parameters were estimated using least-squares regression of calibration plot data. The AIC gives the relative performance of the models and the R^2^ denotes the fit to the validation plots: 1) using ground measured B_P_ and *${\overset{-}{\rho }}_{P}$* and 2) using LiDAR estimated B_P_ and ${\overset{-}{\rho }}_{P}$.

Model type	ACD estimation equation	AIC	1) ground *B*_*P*_ and ${\overset{-}{\rho }}_{P}$	2) LiDAR *B*_*P*_ and ${\overset{-}{\rho }}_{P}$
			*R*^2^	*R*^2^
*Asner and Mascaro’s General Model (AM−GM)*				
All stands	$5.11{H_{L}}^{0.271}{B_{P}}^{0.808}{\overset{-}{\rho }}_{P}$	947.8	0.405	0.179
Sugar maple stands Mixed stands	$2.99{H_{L}}^{0.258}{B_{P}}^{0.991}{\overset{-}{\rho }}_{P}$ $10.1{H_{L}}^{0.258}{B_{P}}^{0.616}{\overset{-}{\rho }}_{P}$	944.4	0.453	0.292
*Individual Tree Based General Model (ITB−GM)*				
All stands	$0.285\;{H_{P}}^{1.24}{B_{P}}^{0.870}{\overset{-}{\rho }}_{P}$	1009.6	−0.111	−0.213
Sugar maple stands Mixed stands	$0.552{{\;H}_{P}}^{1.15}{B_{P}}^{0.729}{\overset{-}{\rho }}_{P}$ $0.314\;{H_{P}}^{1.22}{B_{P}}^{0.867}{\overset{-}{\rho }}_{P}$	1002.5	−0.088	−0.330

**Table 3:** Basal area and wood density estimation equations obtained by least squares regression. Explanatory variables were LiDAR metrics top canopy height (H_L_) and gap fraction (G_L_) and forest type derived from aerial photographs in the sugar maple and mixed stand specific equations. The AIC gives the relative performance of the models and the *R*^2^ denotes the fit to the validation plots.

Response variable	Estimation equations	AIC	*R*^2^
*Basal area*			
*B*_*P*_ (all stands)	14.2 + 0.871 H_L_ − 29.4 G_L_	728.5	0.093
*B*_*P*_ (sugar maple stands) *B*_*P*_ (mixed stands)	4.83 + 1.21 H_L_ − 20.3 G_L_ 12.5 + 1.21 H_L_ − 20.3 G_L_	666.2	0.286
*Volume−weighted mean wood density*			
${\overset{-}{\rho }}_{P}$(all stands)	0.533	−307.0	−0.022
${\overset{-}{\rho }}_{P}$ (sugar maple stands) ${\overset{-}{\rho }}_{P}$(mixed stands)	0.576 0.497	−364.3	0.188

Including forest type into the *B*_*P*_ and *${\overset{-}{\rho }}_{P}$* estimation models led to increased goodness−of−fit (R^2^ rose from 0.09 to 0.29 in the *B*_*P*_ models and from −0.02 to 0.19 in the ${\overset{-}{\rho }}_{P}$ equations; Table 3) and was strongly supported by AIC (*B*_*P*_: Δ = 62.3; ${\overset{-}{\rho }}_{P}$: Δ = 57.3). The % RMSE of the *B*_*P*_ estimator fell from 23.3 to 20.7% and that of ${\overset{-}{\rho }}_{P}$ from 11.7 to 10.4% (Fig 2b). The mixed−forest plots had higher basal area and lower wood density than the sugar maple plots (Fig 1). Incorporating forest type improved overall performance of the AM−GM with the R^2^ rising from 0.18 to 0.29 (RMSE: 20.9 vs. 22.5%), with moderate AIC support (Δ = 3.4).

### Estimating the exponents of individual-tree-based generalised model (ITB−GM)

**Fig 4:**
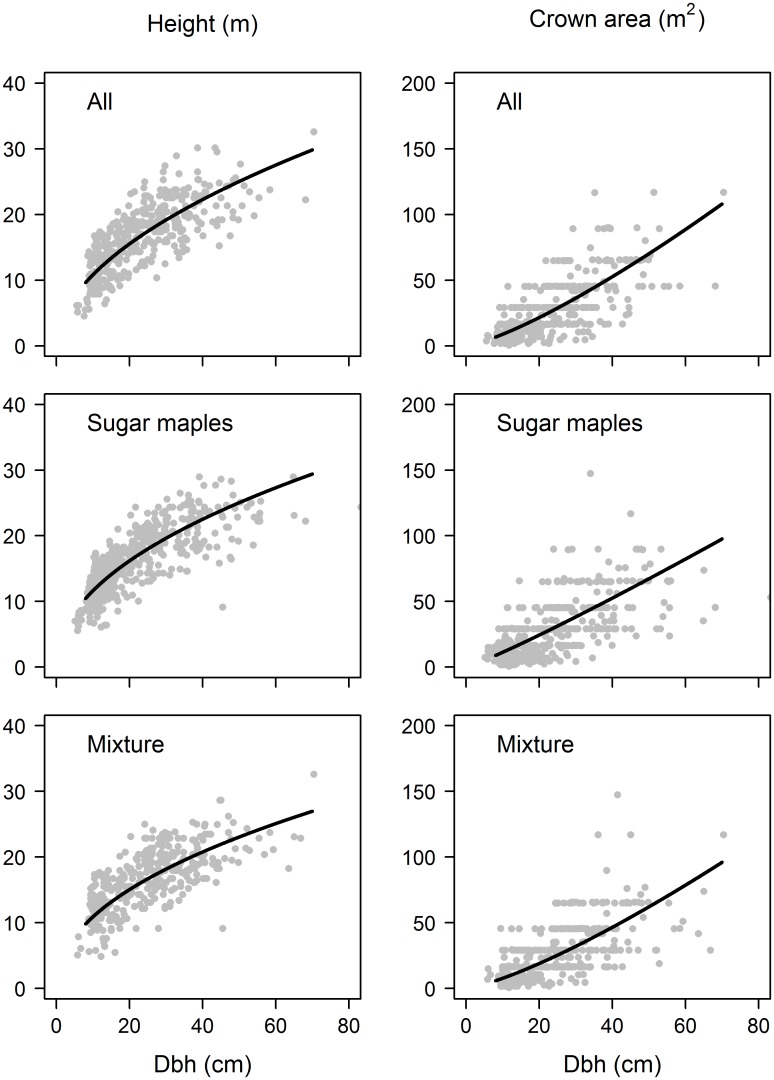
Height−diameter power relationships are given in the left panel whilst the crown area−diameter power relationships are given in the right panel. The exponents from these fitted power functions are used to estimate the powers in the ITB−GM model (Table 4): top row for all stands, middle row for sugar maple stands and the bottom row for mixed stands.

The ITB−GM model, which fixed the values of model parameters based on the field-measured allometries of individual trees, performed less well than the Asner-Mascaro model in which the parameters were estimated by regression. The exponents of ITB−GM estimated from the fitted allometric powers of the H−D and C−D relationships are presented in Table 4 and the fitted relationships are presented in Fig 4. For all stands, height and crown area were fitted as power functions of diameter, with exponents of 0.521 and 1.28 respectively. The log−log regression relationship between summed stem volume $\left(\sum D_{i}^{2+k_{H}}\right)$ and the maximum canopy volume $(\sum D_{i}^{k_{C}+k_{H}})$ had a higher goodness−of−fit (R^2^ = 0.814) than the log−log regression relationship between canopy area (*C*_*P*_) and basal area (*B*_*P*_) (R^2^ = 0.654) indicating that the volume scaling relationship was better supported than the canopy area scaling relationship.

**Table 4:** Estimates of power function parameters of relationships between (a) height vs diameter; (b) crown area vs diameter; (c) summed diameters raised to 2 different powers (see text; crown volume scaling relationship); (d) basal area vs canopy area (canopy area scaling relationship).

Model version	(a) *H*_*i*_ vs *D*_*i*_			(b) *C*_*i*_ vs *D*_*i*_			(c) $\sum D_{i}^{2+k_{H}}$ vs $\sum D_{i}^{k_{C}+k_{H}}$		(d) *C*_*P*_ vs *B*_*P*_	
	*a*_*H*_	*k*_*H*_	*R*^2^	*a*_*C*_	*k*_*C*_	*R*^2^	*k*_*D*_	*R*^2^	*k*_*B*_	*R*^2^
All stands	3.26	0.521	0.593	0.465	1.28	0.419	1.24	0.814	0.701	0.654
Sugar maple stands	3.89	0.476	0.634	0.898	1.10	0.431	1.15	0.659	0.632	0.503
Mixed stands	3.73	0.466	0.503	0.397	1.29	0.378	1.22	0.813	0.711	0.676

**Fig 5:**
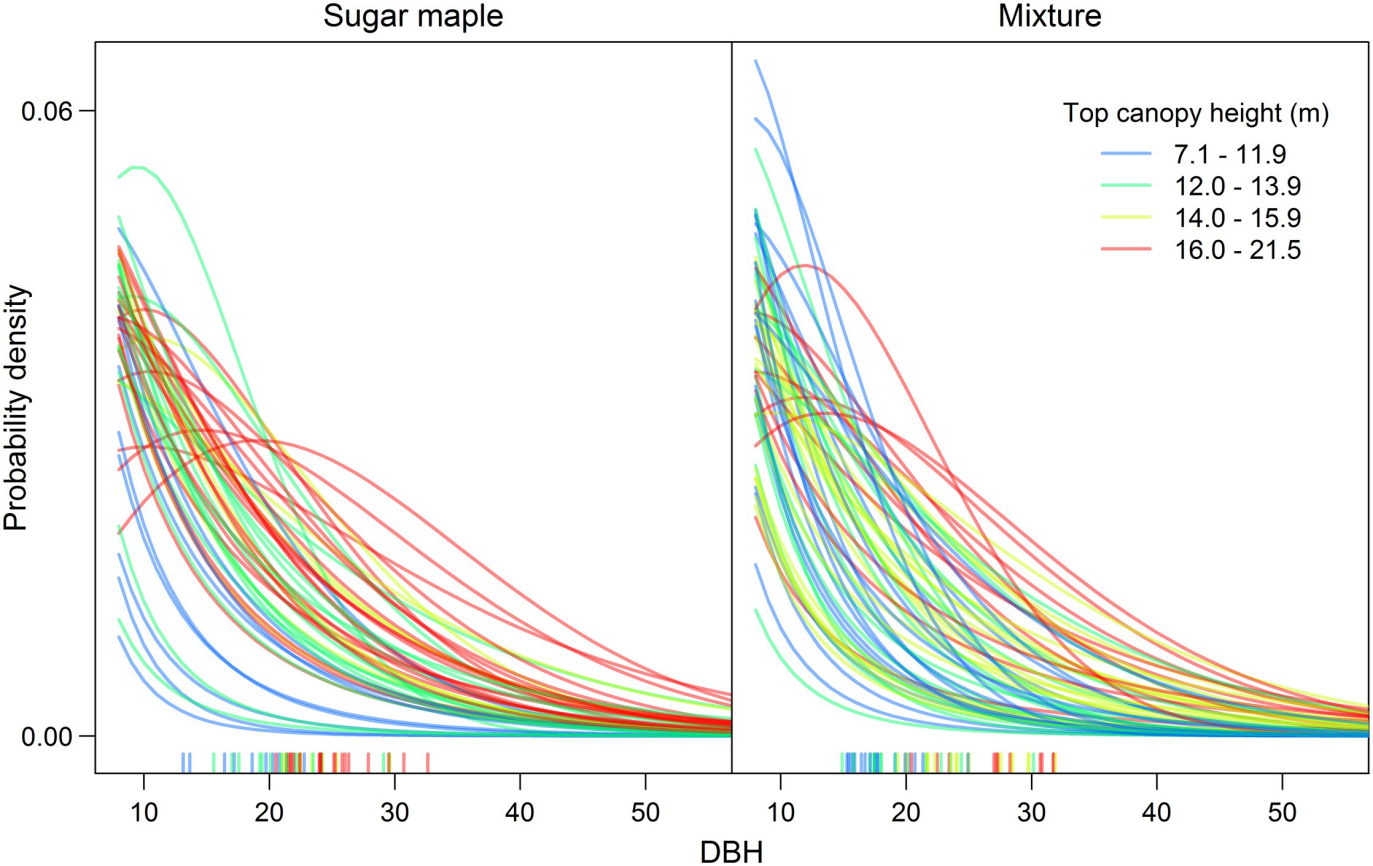
Weibull distributions of tree diameters in each calibration plot. The rug plot along the x-axis shows the quadratic mean diameter of each plot, coloured according to top canopy height. The left panel represents sugar maple stands; the right panel represents mixed stands.

Both scaling relationships contained residual error and had exponent values different from 1 because our set of plots did not follow a single diameter distribution (Fig 5). Although the Weibull distributions that we fit showed that stem diameters were monotonically decreasing in most calibration plots, quadratic mean diameter ranged from 13 to 33 cm across the plots. Plots with a higher QMD generally had a higher top canopy height as measured by LiDAR. In the Supporting Information [S1 Text; S1, S2 and S3 Figs], we provide a comprehensive analysis of how variation in tree diameter distributions affects model fit for a range of different H−D and C−D scaling relationships.

The ITB−GM, with exponents fixed at their theoretical values and *a*_4_ fitted by linear regression is given in Table 2. The exponent associated with *B*_*P*_ was similar in the two models (AM−GM: 0.81 vs ITB−GM: 0.87), but the exponent associated with *H*_*L*_ differed greatly (0.271 vs 1.24). The ITB−GM model had a RMSE of 27.3%, indicating it is less able to explain variance in biomass than the AM−GM (22.5%).

The best predictions were obtained by using the AM−GM and including forest type information (RMSE: sugar maple: 15.8%; mixture: 25.5%). The exponent of the *H*_*L*_ term in the AM−GM was unaffected by forest type, but the *B*_*P*_ exponent of mixed stands was much lower than the sugar maple exponent (0.616 vs 0.991; Table 2). Including forest type led to greater improvements in the fit of the ITB−GM than that of the AM−GM (ΔAIC = 7.1 vs. ΔAIC = 3.4). However, the predictions to the validation plots of the ITB−GM were slightly less accurate (RMSE: no forest types = 27.3%; forest type = 28.6%). In all versions of the model, the sugar maple plots were predicted more accurately than the mixture plots.

## Discussion

Deriving the AM−GM from individual tree measurements has revealed the origins of its parameters, the assumptions behind the power function formula, and the situations in which it is unlikely to make accurate predictions. Below, we explore specific explanations for low goodness−of−fit, including that (1) the basal area and wood density of plots are not closely correlated with top canopy height or gap fraction as measured by LiDAR; (2) tree size distributions are not conserved across the landscape; and (3) the exponents of the allometries are affected by systematic changes in species composition, and the exponent of the crown area allometry deviates from 2. Our findings suggest that among−stand variability in structure and composition are key factors in determining the accuracy of the AM−GM.

### Basal area is weakly correlated with height

Basal area is a key element of allometry-inspired models for estimating forest carbon. It is not directly measured from LiDAR, but instead is inferred indirectly from other height metrics [23]. The goodness−of−fit of the AM−GM for this Canadian forest was substantially reduced when ground−measured *B*_*P*_ and ${\overset{-}{\rho }}_{P}$ were replaced with LiDAR estimates (R^2^ = 0.41 vs 0.18) and therefore LiDAR offered a poor substitute for ground data on these quantities. Predictions of *B*_*P*_ from LiDAR metrics were weak in our study area (R^2^ = 0.09; Fig 1) compared with that reported by Asner et al. [7] for tropical forests (R^2^ ≥ 0.55), although the fit was improved by splitting the plots into two forest types (R^2^ = 0.29). Improving the accuracy of the LiDAR−based models of *B*_*P*_ may therefore require other metrics than *H*_*L*_ and *G*_*L*_ to be included in regression relationships [9, 12] or the application of individual-based approaches [14].

### Tree size distributions vary across the landscape

Basal area is fundamentally linked with the stem diameter distribution, and variability in this distribution weakens the correlation between canopy height and basal area. When the stem diameter distribution follows either a power- or truncated-Weibull function and is conserved across a landscape, then the volume summation and crown area scaling relationships are exact and the exponents of the AM−GM all reduce to 1 [S1 Text; S1 Fig]. However, when the underlying diameter distributions vary among stands, the exponents relating these quantities will deviate from 1 and the accuracy of the relationships will decrease [S3 Fig]. The AM−GM is therefore likely to be less accurate in forests where there is large variability in tree size distributions.

### Why are size distributions more variable in temperate forests than in natural tropical forests?

Size distributions of forests are linked to size−dependent growth and mortality [26], and can be similar across forested landscapes if these demographic functions remain constant over space and time [27, 16]. This may be a reasonable assumption in old-growth tropical forests where size distributions are often close to power functions with exponents of roughly −2 [28] (but see [27]). Temperate forests are often managed and comprise a patchwork of stands at different stages of recovery following disturbance (natural or human). Temperate forest size distributions tend to be more variable [29] and are often modelled by a Weibull distribution with the flexibility to fit both unimodal and power function−type distributions [16]. The selection−managed forests considered here are uneven−aged, and exhibit varying tree size distributions as a legacy of their management history. Our analyses suggest that assumptions of the AM−GM are compromised in structurally heterogeneous forests, and that this model is not expected to produce high goodness−of−fits in such areas. In our particular study area, changing management practices over time have produced a wide range of diameter distributions, which in turn have weakened the accuracy of the AM−GM.

### Wood density is very weakly correlated with LiDAR-measured height

LiDAR and RADAR measure forest structure, but not wood density. Predictive models can give rise to markedly different maps of ACD depending on the assumed spatial variation in wood density [30]. Wood density $\left({\overset{-}{\rho }}_{P}\right)$ was even less well predicted (R^2^ = -0.02) from LiDAR than basal area, but was improved by separating the landscape into forest types (R^2^ = 0.19) because conifer and broadleaf species vary in wood density. There is no evidence in our derivation, or from previous work [10], that ${\overset{-}{\rho }}_{P}$ should have an associated power in the AM−GM, even though the model has commonly been fitted with an ${\overset{-}{\rho }}_{P}$ exponent included [7, 10]. Consistent with theory, we found that including the ${\overset{-}{\rho }}_{P}$ exponent (*b*_*3*_) did not lead to significant improvements in model fit in our temperate data.

### Influences of crown area allometry on goodness of fit

The exponent of the C−D relationship, *k*_*C*_, can also affect accuracy. When *k*_*C*_ = 2, the powers in the ITB−GM all reduce to 1, total stem volume is directly proportional to the maximum canopy volume and canopy area is directly proportional to basal area. The AM−GM is therefore most accurate when *k*_*C*_ = 2; conversely, the further *k*_*C*_ departs from 2, the more inaccurate the volume and crown area summation scaling relationships become [S1 Text; S1 Fig]. Even with variable size distributions, the goodness−of−fit of the total stem volume vs canopy volume relationship is high (R^2^ > 0.8) when *k*_*C*_ is greater than 1.3. There is a sharp drop off in the accuracy of the volume scaling relationship if the C−D exponent is less than 1.3 [S1 Fig], and the AM−GM is expected to perform poorly in forests with variable size distributions when the C−D exponent has a lower value. Since *k*_*C*_ was 1.28 for the Canadian temperate forest, the crown area allometry also contributed to low model accuracy.

We lack a clear picture of how *k*_*C*_ varies globally, but there is some evidence that values are lower for temperate forests. Classical self−thinning theory was based on an assumption of an exponent of 2 [31, 32], whereas metabolic scaling theory predicts an exponent of 4/3 [33], both above the threshold of 1.3 below which accuracy deteriorates. An average value of *k*_*C*_ = 1.36 was found for tropical forests [31], whereas a wide range of *k*_*C*_ values have been reported for temperate forests (0.85 for Virginia, USA, [34]; 1.19 for European beech, [35]; 2.16 for New Zealand mountain beech, [32]). Competition amongst the trees becomes an important feature determining crown shape and the C-D exponent [32] and that too varies at different scales. The goodness−of−fits of the C−D power functions in our analyses were low (R^2^ < 0.45), suggesting that uneven−aged stands may require a variable relationship between height and diameter, which would consequently require an alternative formulation of the AM−GM. Dietze et al. [36] found that the C−D scaling relationship was more variable than the H−D relationship for two managed temperate forest sites in North Carolina, USA.

The H−D scaling exponent, *k*_*H*_, has less influence on the ITB−GM than *k*_*C*_, as it only contributes to the volume scaling relationship and appears on both sides of this equation. The magnitude of *k*_*H*_ affects the accuracy of the power function by influencing the relative magnitude of the summations; increasing *k*_*H*_ would mitigate the effects of *k*_*C*_ deviating from 2 [S1 Fig].

### Influences of forest composition on power-law exponents and goodness of fit

Changes in forest composition within a landscape can have major effects on ACD estimates if those changes are associated with systematic variation in crown geometry and wood density [12, 30]. In our study area, the model was not substantially improved when forest type was accounted for (Fig 3), but an examination of its assumptions highlighted some combinations of H−D and C−D exponents where forest type could influence the generality of the model [S1 Fig]. Given that the AM−GM is based on scaling relationships of individual trees (H−D and C−D), it is clear that species composition may be important if it results in changes to these allometric functions across the landscape. Previous studies indicate that H−D and C−D power functions vary with site and species, suggesting that AM−GM exponents will vary across heterogeneous landscapes. The inclusion of forest type improved the ACD predictions of the sugar maple stands more than the mixed stands. Delineation of the sugar maple forest type, which essentially represents a single species, may therefore have been beneficial because there is expected to be more variation in allometry between species than within species. Lines *et al*. [37] noted that the H−D relationships of Spanish conifer species had exponents close to 2/3 (the value predicted by biomechanical theory), but those of broadleaf species were much more variable and often less than 2/3 [35]. Such differences between conifers and broadleaves could result in different AM−GM exponents across forests with shifting species dominance.

## Conclusion

The allometry-inspired AM-GM model appears to predict forest carbon more reliably in tropical forests than in temperate ones. Asner and Mascaro [8] achieved a goodness−of−fit of R^2^ = 0.83 compared with R^2^ = 0.18 in this study, even though the models were identical (Table 2). Their RMSE was 9% of the mean ACD compares with 23% for our models (Fig 3). Duncanson et al. [14] also observed poor model performance when testing the AM−GM in two out of three temperate forest sites in the USA (R^2^ = 0.13, 0.18 and 0.73).

A key issue is that stand basal area is weakly correlated with canopy height in temperate landscapes comprised of patchworks of stands at various stages of succession/development after disturbance. Selection management created a variety of structural conditions in the Canadian forests studied here, whereas in natural temperate forests variation in stand structure is induced by disturbance from wind, disease, fire and pests. Variability in regeneration, growth and mortality among these stands leads to weak correlations between basal area and height–whereas these are closely coupled in many tropical forests [7]. The allometry-inspired model is reliant on predicting basal area from height, which is a particular problem in heterogeneous landscapes.

Deriving the AM−GM from individual tree information further underscores the importance of variability in size distributions across landscapes. Given that a tree’s biomass is obtained by multiplying its wood volume by its wood density (and assuming conical form), the values of *b*, *c* and *d* in the individual biomass model function $aH_{i}^{b}\;D_{i}^{c}\;\rho_{i}^{d}$ should be close to 1, 2 and 1, respectively [10, 38]. By analogy we would expect *b*_*1*_, *b*_*2*_ and *b*_*3*_ to all be approximately 1 in the AM−GM if the summation had no effect on exponents; however, two of the exponents are far from 1 for the tropical forests analysed by Asner and Mascaro [8] (*b*_*1*_ = 0.28, *b*_*2*_ = 0.97 and *b*_*3*_ = 1.38). Non-linearities in the process of scaling from trees to stands are clearly influential in determining these exponents. This also explains why our ITB-GM was ineffective.

This paper has described the theoretical basis of the AM-GM, demonstrating that the reliability of the approach is dependent on having invariant size distributions across landscapes and on the crown area-diameter power relationship of individual trees. Landscape heterogeneity in these attributes resulted in the poor performance of the AM-GM in a managed temperate system compared with species-rich tropical forests. Model performance is improved by stratification into forest types, but this does not address the issue of varying size distributions. More studies into the spatial variability of tree size distribution are needed to understand when allometry-inspired general models can be reliably used to predict forest aboveground carbon stocks.

## Supporting information

S1 Text Assessing the validity of the volume and canopy area scaling relationships. (DOCX)

S1 Table Species compositions extracted from the 114 calibration plots. Trees from the tree height and crown area dataset were sampled to match these compositions.
(DOCX)

S1 Fig Goodness−of−fit with different values of *k*_*H*_ and *k*_*C*_ when substituting top canopy height (*H*_*L*_) and basal area (*B*_*P*_) into the ITB−GM (5) using the relationships modelled by the volume and canopy area scaling relationships. Parameter values for the modelled relationships are also given. For particular values of *k*_*H*_ and *k*_*C*_, each matrix cell represents a relationship fitted to the 114 calibration plots. The square matrices give the power (*k*_*D*_), coefficient (*a*_*D*_) and R^2^ of the relationship in the volume scaling relationship, whilst the bars give the equivalent (*k*_*B*_, *a*_*B*_ and R^2^) for the canopy area scaling relationship. In the square matrices, both *k*_*H*_ and *k*_*C*_ vary, whilst only the latter affects the bars. Points represent the values of *k*_*H*_ and *k*_*C*_ estimated from allometric data (Table 4).
(TIFF)

S2 Fig Goodness−of−fit of the scaling relationships when underlying size distributions follow a power function or Weibull distribution. The square matrices represent R^2^ values for the volume scaling relationship and the bars represent R^2^ values for the canopy area scaling relationship as the exponent parameters of H−D and C−D are varied. The leftmost and centre panels represent pseudo−data plots that exhibit a power function and a Weibull distribution, respectively. The rightmost panels show the difference in R^2^ for each combination of *k*_*H*_ and *k*_*C*_.
(TIFF)

S3 Fig Exponent values and goodness−of−fit of the volume summation and crown area assumptions as the H−D and C−D relationships are varied and as the Weibull stem diameter distributions become more variable. The exponent of the volume scaling relationship when the Weibull parameters were changed to produce low and high variance is given in the top row. The difference in R^2^ between each of the variable Weibull datasets and the fixed Weibull is given in the bottom matrices, where the square matrices correspond to the volume scaling relationship and the bars correspond to the canopy area scaling relationship.
(TIFF)

S1 Dataset Plot data used for main analyses. (CSV)
