# Supplementary material for: A critique of general allometry-inspired models for estimating forest carbon density from airborne LiDAR
Source: PLoS One. 2019 Apr 19;14(4):e0215238. doi: 10.1371/journal.pone.0215238 (PMC6474603; doi:10.1371/journal.pone.0215238)
Supplement: S1 Text — (DOCX) [file pone.0215238.s001.docx]

# S1 Text

**Assessing the validity of the volume and canopy area scaling relationships**

To estimate the exponents of the AM−GM (5), we needed to estimate $k_{D}$ and $k_{B}$ for given combinations of the H−D and C−D power function exponents ($k_{H}$ and $k_{C}$). To assess model validity for other values of $k_{H}$ and $k_{C}$, we iterated through different combinations of $k_{H}$ (ranging from 0 to 1.5, in increments of 0.03) and $k_{C}$ (ranging from 0 to 2.5 in increments of 0.05). For each combination, the summations in the volume and canopy area scaling relationships were calculated from the 114 calibration plots and then we fit a power function through these data. The coefficients ($a_{D}$ and $a_{B}$), exponents ($k_{D}$ and $k_{B}$) and $R^{2}$ of these two power functions were recorded for each combination of $k_{H}$ and $k_{C}$.

[S1 Fig] presents the results of fitting the power functions in the volume and canopy area scaling relationships to a range of different combinations of $k_{H}$ and $k_{C}$. Higher values of $k_{C}$ corresponded to a better fit in both equations.

**Are the ITB**−**GM assumptions influenced by stem diameter distributions?**

We explored whether the accuracy of either the volume summation or canopy area scaling relationships was influenced by whether the underlying stem diameter distribution followed a power function or Weibull distribution. We generated pseudo−data for 200 plots: 100 following a −2 power function bounded between 8 and 85 cm, and 100 following a Weibull distribution truncated at 8 cm and with a shape parameter of 1.8 and scale parameter of 30. The parameters for the power function distribution were chosen to be more similar to those observed in the tropics and the parameters for the Weibull distribution were chosen to be different from a power function yet still representative of stem diameter distributions in the study area. A set of diameters was obtained from each of these distributions using their inverse cumulative distribution functions and a uniform random number generator. The number of sampled diameters was randomly chosen from a uniform distribution bounded at realistic numbers of stems observed in the calibration plots (50 to 250). We then used the methods described in the previous section for both sets of 100 plots (power function and Weibull distribution plots) and calculated the difference between the $R^{2}$ of the fitted relationships used in the assumptions.

[S2 Fig] compares the effects of different diameter distributions on the accuracy of the volume summation and canopy area scaling relationships. Differences in $R^{2}$ are small overall (< 0.08). However, a Weibull diameter distribution produced marginally more accurate results when the exponent of the C−D relationship is low ($k_{C}$ < 0.5).

**What if the underlying stem diameter distributions followed a power function?**

To further understand the volume summation and canopy area scaling relationships, we replaced each summation with an integral derived from an underlying power function distribution with the following probability density function:

| $p\left( D \right)=\alpha D^{-\beta}$  $\alpha=\frac{1-\beta}{{D_{max}}^{1-\beta}-{D_{min}}^{1-\beta}}$ | (S.1) |
| --- | --- |

where $\alpha$ is a normalising constant such that $p\left( D \right)$ is a valid probability density function, with $D_{min}$ and $D_{max}$ denoting the minimum and maximum diameters. Assuming a list of diameters ($D_{i}$,…$D_{N}$) are drawn from $p\left( D \right)$ where the number of diameters $N$ is large, then a given power function summation can be approximated by:

| $\sum_{i=1}^{N} {D_{i}}^{\gamma}\approx N\int_{D_{min}}^{D_{max}} D^{\gamma}p\left( D \right) dD=\alpha N\int_{D_{min}}^{D_{max}} D^{\gamma-\beta} dD$ | (S.2) |
| --- | --- |

which can be solved to give:

| $\sum_{i=1}^{N} {D_{i}}^{\gamma}\approx\frac{\alpha N}{\gamma-\beta+1}\left[ {D_{max}}^{\gamma-\beta+1}-{D_{min}}^{\gamma-\beta+1} \right]$ | (S.3) |
| --- | --- |

The volume scaling relationship is $\sum D_{i}^{2+k_{H}}\approx a_{D}\left( \sum D_{i}^{k_{C}+k_{H}} \right)^{k_{D}}$, and so using equation (S.3), we can rewrite this as:

| $\frac{\alpha N}{2+k_{H}-\beta+1}\left[ {D_{max}}^{2+k_{H}-\beta+1}-{D_{min}}^{2+k_{H}-\beta+1} \right]\approx a_{D}\left( \frac{\alpha N}{k_{C}+k_{H}-\beta+1}\left[ {D_{max}}^{k_{C}+k_{H}-\beta+1}-{D_{min}}^{k_{C}+k_{H}-\beta+1} \right] \right)^{k_{D}}$ | (S.4) |
| --- | --- |

Assuming that the underlying power function is conserved across all plots, for equation (S.5) to be consistent for all possible numbers of stems across plots, $N$, then it must be the case that $k_{D}$ = 1. Taking the log of both sides of the equation and rearranging gives the solution for $a_{D}$:

| $log(a_{D})\approx\log\left( k_{C}+k_{H}-\beta+1 \right)-log \left( 2+k_{H}-\beta+1 \right)+log({D_{max}}^{2+k_{H}-\beta+1}-{D_{min}}^{2+k_{H}-\beta+1})-log({D_{max}}^{k_{C}+k_{H}-\beta+1}-{D_{min}}^{k_{C}+k_{H}-\beta+1})$ | (S.5) |
| --- | --- |

Therefore, the volume summation scaling relationship is mathematically valid when stem diameters follow a common power function distribution.

**What if the underlying stem diameter distributions followed a Weibull distribution?**

The same process can be repeated for a truncated Weibull distribution with a probability density function:

| $p\left( D \right)=\frac{k}{\lambda}\left( \frac{D}{\lambda} \right)^{k-1}exp\left( \left( \frac{D_{min}}{\lambda} \right)^{k}-\left( \frac{D}{\lambda} \right)^{k} \right)$ | (S.6) |
| --- | --- |

where $k$ and $\lambda$ are the shape and scale parameters. For a given set of diameters, the integral is:

| $\sum_{i=1}^{N} {D_{i}}^{\gamma}\approx\frac{Nk}{\lambda^{k}}\int_{D_{min}}^{D_{max}} D^{\gamma+k-1}exp\left( \left( \frac{D_{min}}{\lambda} \right)^{k}-\left( \frac{D}{\lambda} \right)^{k} \right) dD$ | (S.7) |
| --- | --- |

which can be solved to give:

| $\sum_{i=1}^{N} {D_{i}}^{\gamma}\approx N\lambda^{\gamma}exp\left( \left( \frac{D_{min}}{\lambda} \right)^{k} \right)\left[ Г\left( \frac{\gamma+k}{k},D^{k}\lambda^{-k} \right) \right]_{D_{min}}^{D_{max}}$ | (S.8) |
| --- | --- |

where $Г$ denotes the lower incomplete gamma function. Substituting this solution into the volume scaling relationship, taking the log of both sides and rearranging as before gives the corresponding solution for $a_{D}$:

| $\log\left( a_{D} \right)\approx\left( 2-k_{C} \right)\log\left( \lambda\right)+\log\left( \left[ Г\left( \frac{(2+k_{H}+k}{k},D^{k}\lambda^{-k} \right) \right]_{D_{min}}^{D_{max}} \right)-\log\left( \left[ Г\left( \frac{(k_{C}+k_{H}+k}{k},D^{k}\lambda^{-k} \right) \right]_{D_{min}}^{D_{max}} \right)$ | (S.9) |
| --- | --- |

The volume scaling relationship is therefore also mathematically valid when stem diameter distributions follow a common Weibull distribution. Taking the same approach for the canopy area scaling relationship demonstrates that it is also valid provided that the stem diameter distribution, either a power function or Weibull, is conserved. The powers in the AM−GM should all equal 1 in this case.

**How are inaccuracies introduced into the assumptions?**

The volume summation and crown area assumptions are accurate when the underlying stem diameter distributions follow the exact same power function or truncated Weibull distribution with no effect from variable stem numbers across plots. The powers in the AM−GM are only necessary when the stem diameters deviate from these fixed distributions. To examine this effect, we generated pseudo−data for three sets of 100 plots, as detailed before, where each set was based on a Weibull distribution with: (a) the parameter values fixed as before, i.e. no variance in the parameters ($k$ = 1.8 and $\lambda$ = 30), (b) low variance in the parameter values (1.6 ≤ $k$ ≤ 2 and $\lambda$ = −6.39 + 17.3$k$ + Ɲ(0, 3.48); $\lambda$ is linearly related to $k$ in the calibration plots where the error term is drawn from a normal distribution with mean 0 and standard deviation 3.48) and (c) high variance in the parameter values (simulating the variance observed in the calibration data: 0.5 ≤ $k$ ≤ 2 and $\lambda$ = −6.39 + 17.3$k$ + Ɲ(0, 3.48)). We recorded the exponent of the volume scaling relationship for the (b) and (c) datasets at different combinations of $k_{C}$ and $k_{H}$ and then compared the $R^{2}$ of the power function relationships of (a) vs (b) and (a) vs (c).

The power of the volume scaling relationship only deviated slightly from 1 when there was low variance in the parameters of the underlying Weibull distributions whereas the power deviated substantially more when there was higher variance [S3 Fig]. The $R^{2}$ values of the relationship also decreased as the Weibull distributions became more variable, with the strongest differences occurring under a combination of low $k_{C}$ and $k_{H}$ values.
