# Supplementary material for: A critique of general allometry-inspired models for estimating forest carbon density from airborne LiDAR
Source: PLoS One. 2019 Apr 19;14(4):e0215238. doi: 10.1371/journal.pone.0215238 (PMC6474603; doi:10.1371/journal.pone.0215238)
Supplement: S1 Table — Trees from the tree height and crown area dataset were sampled to match these compositions. (DOCX) [file pone.0215238.s002.docx]

**Table S.1** Species compositions extracted from the 114 calibration plots. Trees from the tree height and crown area dataset were sampled to match these compositions.

| **Species** | **All stands** | **Sugar maple stands** | **Mixed**  **Stands** |
| --- | --- | --- | --- |
| Sugar maple  *Acer saccharum* (Marsh.) | 0.232 | 0.437 | 0.105 |
| American beech  *Fagus grandifolia* Ehrh. | 0.110 | 0.133 | 0.096 |
| Eastern hemlock  *Tsuga canadensis* (L.) Carrière | 0.141 | 0.045 | 0.200 |
| Yellow birch  *Betula alleghaniensis* Britt. | 0.055 | 0.061 | 0.051 |
| Red maple  *Acer rubrum* (L.) | 0.097 | 0.049 | 0.128 |
| Balsam fir  *Abies balsamea* (L.) Mill. | 0.168 | 0.049 | 0.242 |
| White spruce  *Picea glauca* (Moench) Voss | 0.029 | 0.008 | 0.042 |
| Conifers | 0.018 | 0.010 | 0.023 |
| Broadleaves | 0.150 | 0.208 | 0.113 |
