# Supplementary figures and images for: A critique of general allometry-inspired models for estimating forest carbon density from airborne LiDAR
Source: PLoS One. 2019 Apr 19;14(4):e0215238. doi: 10.1371/journal.pone.0215238 (PMC6474603; doi:10.1371/journal.pone.0215238)

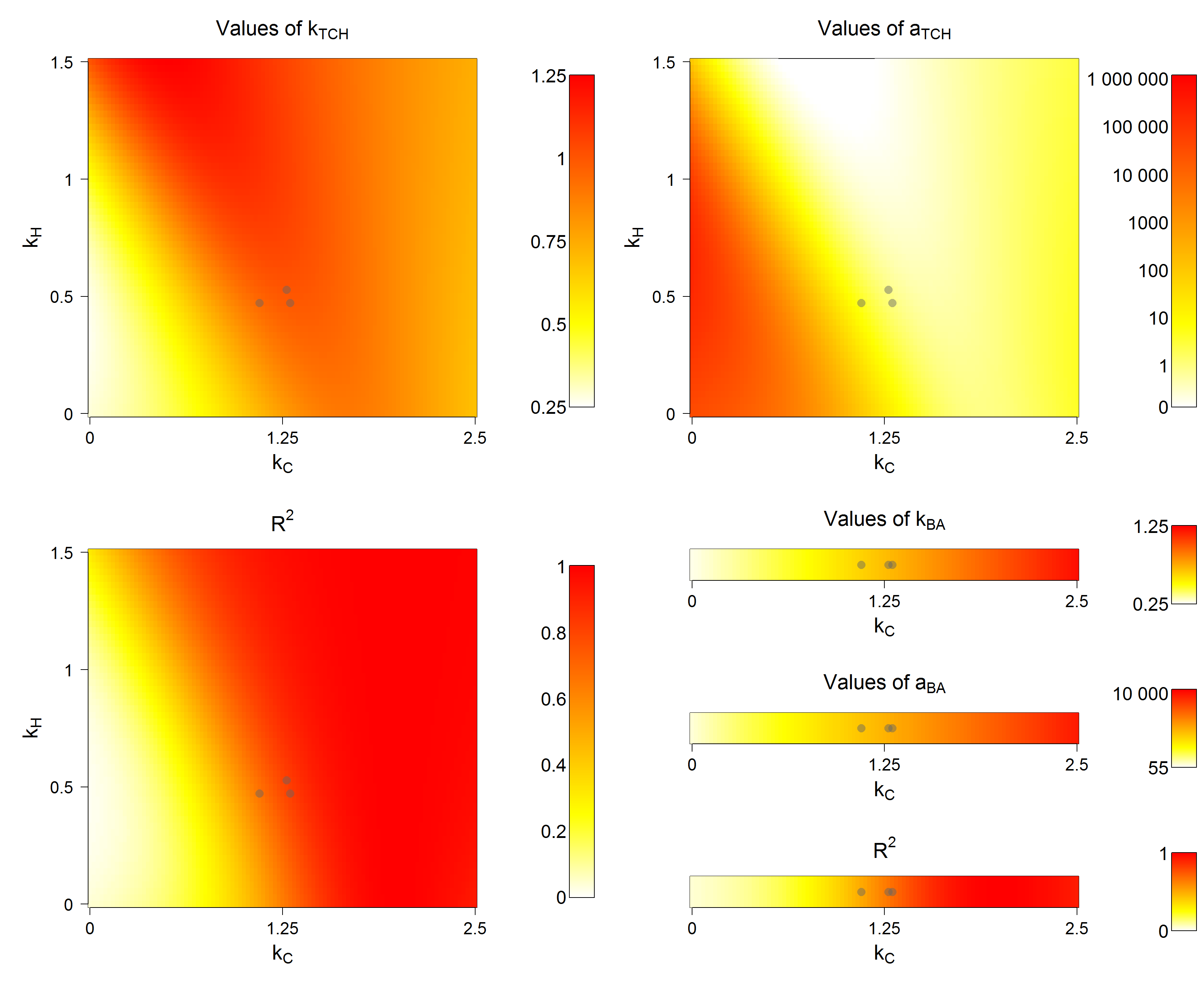

Supplement: S1 Fig — Parameter values for the modelled relationships are also given. For particular values of kH and kC, each matrix cell represents a relationship fitted to the 114 calibration plots. The square matrices give the power (kD), coefficient (aD) and R2 of the relationship in the volume scaling relationship, whilst the bars give the equivalent (kB, aB and R2) for the canopy area scaling relationship. In the square matrices, both kH and kC vary, whilst only the latter affects the bars. Points represent the values of kH and kC estimated from allometric data (Table 4). (TIFF) [file pone.0215238.s003.tiff]

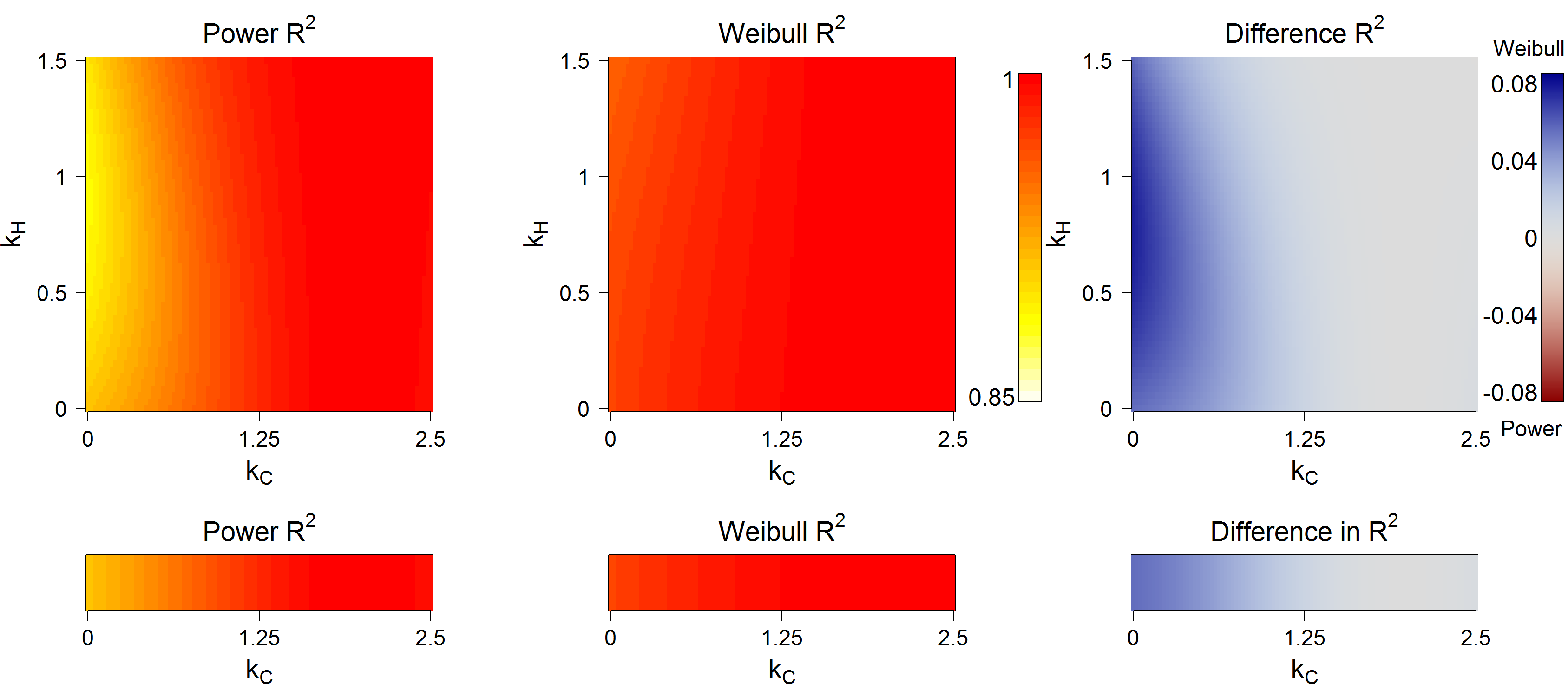

Supplement: S2 Fig — The square matrices represent R2 values for the volume scaling relationship and the bars represent R2 values for the canopy area scaling relationship as the exponent parameters of H−D and C−D are varied. The leftmost and centre panels represent pseudo−data plots that exhibit a power function and a Weibull distribution, respectively. The rightmost panels show the difference in R2 for each combination of kH and kC. (TIFF) [file pone.0215238.s004.tiff]

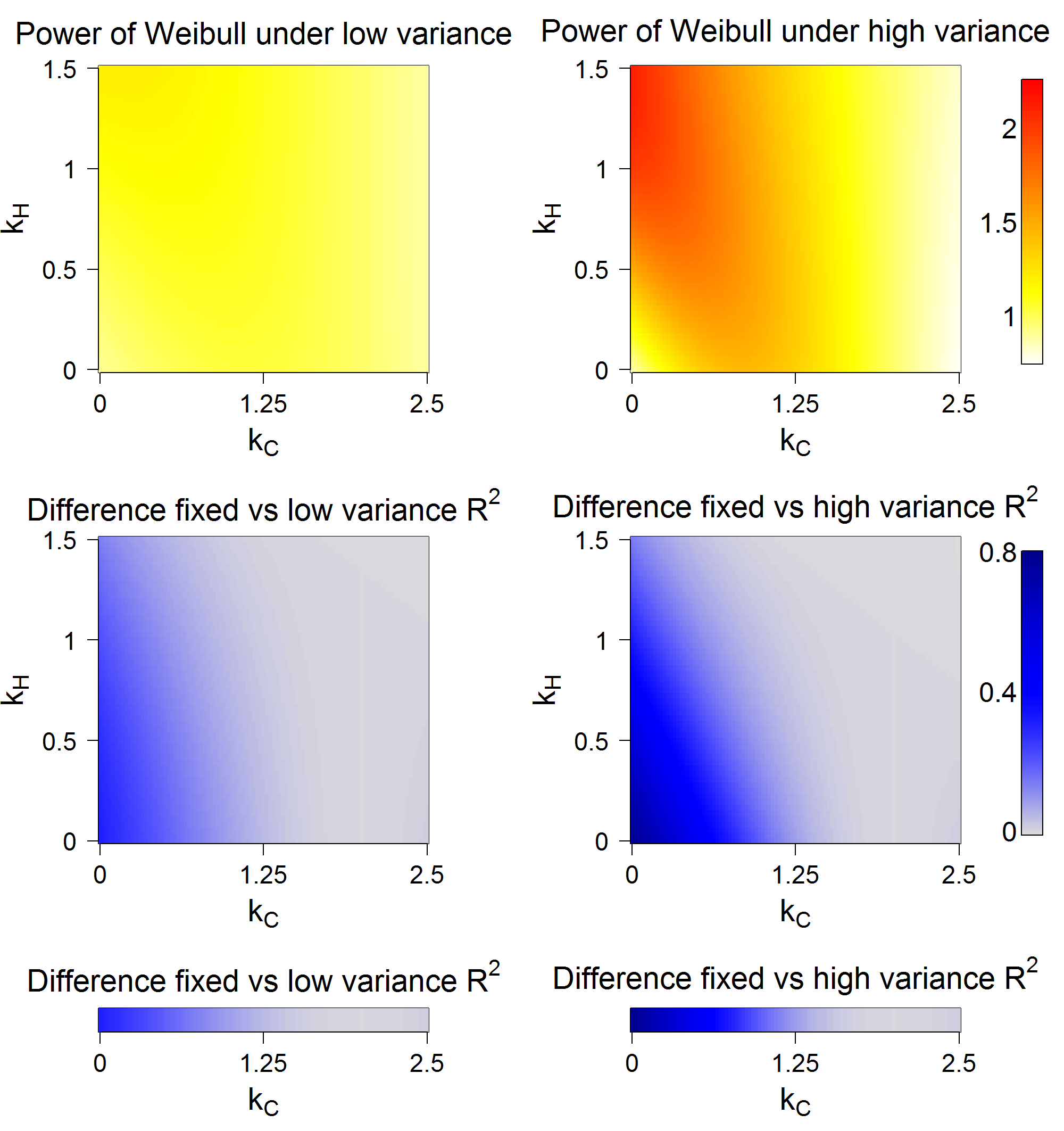

Supplement: S3 Fig — The exponent of the volume scaling relationship when the Weibull parameters were changed to produce low and high variance is given in the top row. The difference in R2 between each of the variable Weibull datasets and the fixed Weibull is given in the bottom matrices, where the square matrices correspond to the volume scaling relationship and the bars correspond to the canopy area scaling relationship. (TIFF) [file pone.0215238.s005.tiff]
